# Intraoperative parathormone increase after focused parathyroidectomy in a patient with sarcoidosis – Case report

**DOI:** 10.1016/j.amsu.2021.102577

**Published:** 2021-07-17

**Authors:** Nina Pislar, Marko Hocevar

**Affiliations:** Department of Surgical Oncology, Institute of Oncology Ljubljana, Zaloska Cesta 2, 1000, Ljubljana, Slovenia

**Keywords:** Primary hyperparathyroidism, Focused parathyroidectomy, Hypercalcaemia, Wisconsin criteria, Case report

## Abstract

**Introduction:**

Hypercalcaemia is most commonly a sign of primary hyperparathyroidism but can also be a sign of an active granulomatous disease. Standard treatment for primary hyperparathyroidism caused by a solitary parathyroid gland adenoma identified by localisation studies is minimally invasive focused parathyroidectomy. If unsuccessful, bilateral neck exploration is recommended.

**Case presentation:**

We report the case of hypercalcaemia and ostheoporosis in a 63–year -old woman with a history of sarcoidosis and suspected primary hyperparathyroidism. Localisation studies for parathyroid adenoma were inconclusive due to active cervical and mediastinal granulomatous lymph nodes. Sarcoidosis was treated with corticosteroids but hypercalcaemia persisted. Focused parathyroidectomy was attempted with intraoperative parathyroid hormone measurement but an increase in parathyroid hormone levels was observed. However, with high clinical probability of a successfully removed adenoma and frozen section evaluation, we decided not to proceed with bilateral neck exploration. Serum parathyroid hormone and calcium levels dropped accordingly the following day.

**Clinical discussion:**

We explored all possible underlying mechanisms for persistent elevated parathyroid hormone level described in literature.

**Conclusion:**

We conclude that Wisconsin Criteria with intraoperative parathyroid hormone measured 20 minutes after adenoma removal should be applied in such cases.

## Highlights

• Primary hyperparathyroidism (PHP) and sarcoidosis can both cause hypercalcaemia. Other possible mechanisms also contribute and can present a diagnostic and therapeutic challenge.
• When Miami criterion fails, it is not always necessary to proceed with bilateral neck exploration. Wisconsin criteria can be used.

## 1 Introduction

Primary hyperparathyroidism (PHP) and granulomatous diseases are the most common causes of hypercalcaemia in adults, aside from malignancy. The underlying mechanism in PHP is autonomous oversecretion of parathyroid hormone (PTH) in most cases due tosolitary adenoma of the parathyroid [1]. Other mechanisms of hypercalcaemia are PTH-related peptide (PTH-rp) secretion typical for neoplasms, Vitamin D overproduction and excretion of other growth factors or cytokines [2].

Surgery offers complete cure in most cases of PHP, with focused parathyroidectomy and intraoperative PTH (ioPTH) measurement as the procedure of choice. With the use of 18F-fluorocholine PET-CT (FCh PET) for preoperative localisation and multiglandular disease exclusion, ioPTH is no longer necessary [4]. If this approach is unsuccessful, traditional bilateral neck exploration with higher complication and morbidity rates is indicated [5].

Sarcoidosis is a benign granulomatous disease that presents with hypercalcaemia in 10% of cases [6]. The usual cause is Vitamin D overproduction [2], but rare cases of PTH-rp mediated hypercalcaemia have been described [7,8].

We present an interesting case of hypercalcemia in a patient with sarcoidosis and suspected PHP. The case report has been prepared in line with the SCARE 2020 Criteria [3].

## 2 Presentation of case

A 63 year old woman was referred to our outpatient clinic by an endocrinologist after PHP was suspected following an osteoporotic fracture. During follow-up visits at the orthopaedic clinic, hypercalcaemia was observed and identification of the underlying cause was required. She was diagnosed and treated for sarcoidosis 14 years ago but has been in remission since. She had no co-morbidities, was not taking any medication and did not report any allergies. Her family history was not significant for any metabolic disease.

Serum calcium concentration was 2.77 mmol/L (reference range 2.15–2.55 mmol/L), phosphate 1.01 mmol/L (reference range 0.81–1.45 mmol/L) and magnesium 0.89 mmol/L (reference range 0.7–1.05 mmol/L). Creatinine and urea levels were normal, 57 μmol/L (reference range 54–84 μmol/L) and 4.5 mmol/L (reference range 2.8–8.1 mmol/L), respectively. 24-hour calcium urine level was 13.2 mmol (reference range 2.5–7.5 mmol/L). Plasma intact PTH (iPTH) level was 161 ng/L (reference range 15–65 ng/L), and 25-hydroxyvitamin D was 22.9 nmol/L (normal above 50 nmol/L). Bone mineral density was decreased (T-value femoral neck −3.0 SD, distal radius −4.5 SD).

A neck ultrasound showed enlarged supraclavicular and jugular lymph nodes without clearly enlarged parathyroid glands. FCh PET showed metabolic activity enhancement (SUV max 11.5) below lower left thyroid lobe, consistent with a 0.9 × 5.6 cm soft tissue mass on CT. Comparable metabolic activity was seen in the right mediastinal (SUV max 12.5), supraclavicular, pectoral and axillary lymph nodes (comparable SUV max). With multiple foci of increased uptake on FCh PET and a history of sarcoidosis, active granulomatosis was suspected. Her chitotriosidase level was elevated at 2304 nmol/ml/h, while ACE level was within normal range (0.31 nmol/ml/min). She was referred to a pulmonologist.

Bronchoscopy and biopsy confirmed active pulmonary granulomatosis. She was started on inhalatory corticosteroids for bronchial sarcoidosis and denosumab for hypercalcaemia. Because of low vitamin D levels, supplementation with 1000 IU/d was started.

At the six month follow-up visit, PTH levels had decreased, but were still above normal: iPTH 106 ng/L, phosphate 0.78 mmol/L, magnesium 0.88 mmol/L. Hypercalcaemia worsened despite an achieved remission of sarcoidosis with calcium levels of 2.86 mmol/L. The patient also had osteoporosis, so surgical treatment of PHP was suggested. Although on previous FCh PET lower left parathyroid gland adenoma was highly likely but the results were inconclusive because of metabolically active granulomas, we decided not to repeat FCh PET to avoid patient radiation exposure. Instead, we opted for focused parathyroidectomy with ioPTH measurement under general anaesthesia.

A lower left parathyroid adenoma (2.5 x 1.5 × 0.8 cm, 2.98 g) was identified and removed with a 2 cm central neck incision and the lateral trap door approach. A senior consultant surgeon specialized in endocrine surgery performed the procedure. iPTH was measured after general anaesthesia induction and 10 minutes after adenoma removal according to the Miami Criterion. PTH levels increased from 103 ng/L to 122 ng/L (Table 1). In addition, an intraoperative frozen section was performed and parathyroid adenoma was confirmed by pathologists. It was decided not to proceed with bilateral neck exploration but to finish the operation and check calcium and PTH levels again the next morning.

**Table 1:** Laboratory values. iPTH - intact parathyroid hormone, PTH-rp - parathyroid hormone related peptide.

	iPTH ng/L	Ca mmol/L	PTH-rp (<13.9 pg/ml)
Before surgery	106	2,86	/
Intraoperatively sample 1	103		<7.4
Intraoperatively sample 2	122		<7.4
Day after surgery	12,1	2,47	<7.4

The postoperative course was uneventful and laboratory results 20 hours after surgery were as follows: plasma iPTH 12.1 ng/L, serum calcium 2.47 mmol/L, serum phosphate 1.52 mmol/L, serum magnesium 0.85 mmol/L. The patient recovered well and was discharged the next day with analgesics. At the follow-up visit after nine days she did not report any new symptoms. According to the results (Table 1), ioPTH measurement proved to be a “false negative” and the intraoperative decision to stick to the original plan of focused approach proved right.

## 3 Discussion

Sometimes it is difficult to identify the sole cause of hypercalcaemia and often the causes are multiple [9]. In addition to autonomous oversecretion of PTH, dehydration, renal insufficiency, and Vitamin D hypervitaminosis also contribute to increased calcium concentration. In a case series of 50 patients with retrospectively identified coexisting sarcoidosis and PHP, no differences in calcium, phosphate or PTH levels were observed regardless of which of the two conditions was recognised and treated first [10].

At our institution, focused parathyroidectomy is the treatment of choice for PHP in patients with a solitary adenoma identified on localisation studies. According to the literature ioPTH should be used during the focused approach to exclude multiglandular disease. The Miami Criterion is used by the majority of endocrine surgeons. It is highly accurate (98%) with a low false negative rate (2%) [11]. Surgery is deemed successful if more than a 50% decrease in iPTH is observed from the induction of anaesthesia to 5–10 minutes after adenoma removal. This indicates a surgical cure and predicts postoperative normocalcaemia. If an insufficient decrease or an increase in ioPTH is seen, it is advised to proceed with bilateral neck exploration. This is a traditional surgical treatment for PHP with an increased risk of postoperative complications, longer recovery time and a comparable risk of PHP recurrence [5].

Vitamin D status in the body is usually reported as 25-hydroxivitamin D (25-OH vit D) level. It is biologically inactive but represents the majority of circulating Vitamin D in the body. It is converted to 1,25-dyhydroxyvitamin D (1,25-OH vit D) in the kidney by alpha-hydroxilase, whose activity is highly sensitive to regulatory mechanisms (calcium, phosphate, and PTH). 1,25-OH vit D is the biologically active form of Vitamin D with a short half-life [12]. In sarcoidosis, the most recognised mechanism for hypercalcaemia is increased 1,25-OH vit D concentration due to excessive autonomous action of alpha-hydroxilase produced by alveolar macrophages in sarcoid granulomas. It leads to increased dietary calcium absorption and decreased plasma PTH [6]. Despite initially decreased Vitamin D in our patient, Vitamin D-related hypercalcaemia cannot be excluded with certainty, as the reported levels were of 25-OH vit D and not of 1,25-OH vit D, the active form produced by alveolar macrophages. However, this would not likely influence ioPTH measurement, as sarcoidosis had already been treated and remission reached before surgery.

An alternative mechanism for hypercalcaemia in sarcoidosis mediated via PTH-rp has been described [7,8]. PTH-rp is usually responsible for hypercalcaemia caused by neoplasms and its actions mimic those of PTH [2,13]. We observed a drop in PTH (from 161 ng/L to 106 ng/L) after treating sarcoidosis, but no drop in intraoperative measurements, which led us to explore PTH-rp as the possible cause for hypercalcaemia.

PTH-rp was measured in stored serum from before surgery and from intraoperative samples but levels were low. Granuloma tissue and parathyroid adenoma tissue were stained for PTH, but the immunohistochemical reaction was negative in granuloma tissue and positive in parathyroid adenoma tissue [14]. Thus, PTH-rp was ruled out as an interfering factor in our patient.

Another explanation for the “false negative” ioPTH are nonspecific proteins, which could interfere with ioPTH assay, but this is unlikely because PTH levels dropped to 12.1 ng/L 20 hours after adenoma removal.

An increase in ioPTH after focused parathyroidectomy often raises the suspicion of a multiglandular disease. These patients experience an elevation in ioPTH level 5 minutes after adenoma removal.

The Wisconsin Criteria has been proposed as a guideline in such cases. It suggests that elevated ioPTH 5 minutes after adenoma removal is set as the new baseline value. An additional ioPTH sample should be drawn at 10–15 minutes after adenoma removal and a 50% fall from the new baseline value is expected. If unsuccessful, another sample is drawn 20 minutes after adenoma removal. If ioPTH remains elevated, bilateral neck exploration is indicated [15].

The most likely cause for elevated ioPTH in our patient 10 minutes after adenoma removal was surgical manipulation of the hyperfunctioning parathyroid gland. The Wisconsin Criteria would apply in this case. Thus, the decision to end the procedure was correct in our case, but an additional ioPTH measurement should have been taken 20 minutes after adenoma removal to be certain about not proceeding with bilateral neck exploration.

## 4 Conclusion

For patients with PHP caused by a solitary parathyroid adenoma identified in preoperative localisation studies with a negative ioPTH according to the Miami Criterion, an additional ioPTH measurement 20 minutes after adenoma removal is indicated, instead of proceeding to bilateral neck exploration.

## Ethical approval

None.

## Author contribution

Nina Pislar: data collection, interpretation, paper preparation.

Marko Hocevar: concept and design, data interpretation, paper overview.

## Sources of funding

None.

## Consent

A written informed consent was obtained from the patient for the publication of this case report. A copy of the written consent form is available for review by the Editor-in-Chief of this journal on request.

## Research registration

N/a.

## Provenance and peer review

Not commissioned, externally peer-reviewed.

## Guarantor

Nina Pislar, Marko Hocevar.

## Declaration of competing interest

None declared.
